# Geographical accessibility to smoking cessation treatment facilities across 335 medical areas in Japan: A nationwide cross-sectional descriptive study using large-scale geospatial data

**DOI:** 10.18332/tid/222677

**Published:** 2026-07-18

**Authors:** Yuki Egashira, Ryo Watanabe

**Affiliations:** 1 School of Health Innovation, Kanagawa University of Human Services, Kanagawa, Japan

**Keywords:** smoking cessation, geographical accessibility, spatial analysis, WHO FCTC, Japan

## Abstract

**INTRODUCTION:**

Geographical research on tobacco control has focused predominantly on tobacco retail environments, whereas spatial accessibility studies using methods such as Enhanced Two-Step Floating Catchment Area (E2SFCA) have examined primary care but rarely smoking cessation services. This study aimed to evaluate the geographical accessibility of smoking cessation clinics and hospitals across Japan’s secondary medical areas (SMAs) in a cross-sectional ecological spatial analysis using large-scale spatial data.

**METHODS:**

Accessibility scores (AS) were calculated at 422804 grids nationwide using E2SFCA. Demand was defined as the smoking rate-adjusted population, and supply comprised approximately 19000 facilities registered under Japan’s insurance-covered smoking cessation program. A regional typology was developed using Ward’s hierarchical cluster analysis. The distribution of standardized claim ratios (SCRs) from nationwide claims data (2018–2022) was examined descriptively across clusters.

**RESULTS:**

Of the 335 SMAs, 319 were analyzed after excluding 16 areas with missing SCR data. The median AS varied substantially across areas (range: 286–5626). Three clusters emerged: Cluster 1, ‘Low accessibility–High disparity’ (24.1%); Cluster 2, ‘Moderate accessibility–Low disparity’ (62.4%); and Cluster 3, ‘High accessibility–Low disparity’ (13.5%). Clusters 2 and 3 showed similar Gini coefficients, differing primarily in AS levels. Cluster 3 showed descriptively higher SCR values (median: 117) compared with Clusters 1 and 2 (median: 93–95).

**CONCLUSIONS:**

This study demonstrates substantial geographical disparities in the accessibility of smoking cessation services in Japan. Regions with higher accessibility tended to exhibit lower intra-regional disparities. For the implementation of the WHO FCTC Article 14, ensuring an equitable geographical distribution of services may be as important as expanding the overall number of facilities.

## INTRODUCTION

Smoking is the leading preventable cause of death worldwide, contributing to approximately seven million deaths annually^1^. Article 14 of the WHO FCTC requires parties to provide smoking cessation treatment; however, implementation remains limited globally, with only 26% of countries providing specialized cessation services^2^.

Sociodemographic factors have been associated with disparities in access to smoking cessation treatment, including lower cessation medication prescription rates among women and older adults^3^, the need for sex-specific cessation support^4^, and support disparities among socioeconomically disadvantaged smokers and minorities^5^.

Geographical approaches to tobacco research have primarily focused on tobacco retail environments. A systematic review of 40 studies reported that higher retail outlet density is associated with higher smoking rates and lower quitting rates^6^. Higher retail outlet density has also been linked to smoking initiation^7^, and greater proximity to retail outlets has been associated with reduced maintenance of smoking cessation^8^. Still, the symmetrical question of access to smoking cessation treatment facilities has not been adequately examined.

Research on Geographical access to smoking cessation treatment itself remains limited. Studies conducted outside Japan have reported lower utilization rates of cessation treatment in rural areas^9^, and the association between distance to cessation classes and participation rates^10^. To date, nationwide regional-level assessments of geographical accessibility to cessation treatment have not been conducted in Japan.

Unlike simple facility counts or nearest distance measures, the E2SFCA method jointly accounts for the supply–demand balance and distance decay within the catchment, thereby providing a more realistic representation of spatial accessibility. The E2SFCA method has been widely used to evaluate healthcare accessibility, with studies conducted in various countries targeting primary care and hospitals^11-13^. Yet, to our knowledge, we could not find studies that specifically evaluated geographical accessibility to smoking cessation treatment services.

In Japan, healthcare planning is organized around secondary medical areas, which form the basic geographical framework for service provision. The adult smoking rate in Japan has declined over recent decades but remains a major public health concern, with current smoking prevalence substantially higher among men than women. Within this context, ensuring geographical access to smoking cessation treatment is relevant to national tobacco control policy.

In Japan, insurance coverage for smoking cessation treatment began in 2006, and 15139 facilities nationwide have been registered for the program. However, to our knowledge, no study has evaluated the geographical accessibility of these facilities on a national scale.

This study aimed to evaluate accessibility to smoking cessation clinics and hospitals across Japan’s secondary medical areas (administrative units that serve as the basic framework for healthcare planning) using the E2SFCA method, quantify regional disparities, develop a regional typology, and descriptively examine the distribution of smoking cessation treatment utilization across clusters.

## METHODS

### Study design and setting

This cross-sectional, ecological, spatial analysis included 335 secondary medical areas in Japan. Secondary medical areas are administrative units with populations ranging from approximately 100000 to several million people, and serve as the basic framework for healthcare planning in Japan. This analysis targeted medical facilities registered to provide nicotine dependence management (e.g. smoking cessation clinics and hospitals). All data used in this study were obtained from publicly available secondary sources, including census-based mesh population data, facility notification information, road network data, and nationwide claims data; no primary data collection or self-reported individual data were involved. The population and facility data referred to 2025, the claims data to the period 2018–2022, and the facility notification information to 2025.

### Data sources

*Demand*

We used 500 m mesh population data by sex and age group for 2025, estimated based on the 2020 National Census^14^. To calculate the estimated number of smokers as a potential demand for smoking cessation clinics, we applied sex- and age-specific smoking rates from the sex- and age-specific populations in each 500 m mesh^15^. National sex- and age-specific smoking rates were applied uniformly to the mesh population, because reliable smoking-prevalence estimates at this fine spatial resolution are not consistently available at the regional level. The implications of this assumption are addressed in the Limitations.

*Supply*

Medical facilities registered for nicotine dependence management were extracted from the notification information submitted to the Regional Bureau of Health and Welfare^16-23^. The location information of each facility was obtained by geocoding the addresses from the notification information, 15139 facilities were identified nationwide. The supply capacity for each facility was assigned different weights based on the number of physicians engaged in smoking cessation treatment by facility type (hospitals: mean 2.57 physicians; clinics: mean 1.50 physicians), as reported in a previous study^24^. The average number of physicians by facility type was used as a practical proxy for relative supply capacity, because facility-level data on the actual volume of cessation services are not available in the public notification information. This approach assumes a broadly comparable contribution per physician; its potential to overestimate the effective availability of cessation services is addressed in the Limitations.

*Transportation*

We used nationwide road network data from OpenStreetMap (OSM)^25^. The OSM has been used in previous healthcare accessibility research^26^ and is a reliable source of data. Road network data were constructed as a network dataset to calculate the actual travel time along roads. Travel speeds were set according to the Densely Inhabited District (DID) status and road type based on the National Road and Street Traffic Conditions Survey^27,28^. The modes of transportation were automobiles and walking.

*Treatment utilization*

Standardized claim ratios (SCRs) for nicotine dependence management in secondary medical areas were obtained from NDB Data (National Database of Health Insurance Claims: Japan’s nationwide claims database)^29^. Data from five years (2018–2022) were used, and the mean SCR for each year was calculated. Medical areas with missing data for any year were excluded from analysis (n=16). SCR represents the ratio of observed to expected claims adjusted for population composition; values exceeding 100 indicate utilization higher than the national average. The SCR is aggregated based on facility location, and claim counts include not only initial visits but also subsequent continuation visits.

### E2SFCA method

The E2SFCA method is a two-step approach that evaluates the supply and demand with transportation like travel time, which has advantages compared to a conventional supply–demand study^11^. Following the E2SFCA framework, the catchment area was divided into three equal travel-time zones at 15-minute intervals (0–15, 15–30, and 30–45 minutes). This three-zone structure with 15-minute intervals follows previous E2SFCA-based accessibility studies^30^. Distance-decay weights were then applied to each zone (0–15 min, 1.00; 15–30 min, 0.68; 30–45 min, 0.22). These weights correspond to the slower decay weight set proposed in the original E2SFCA study by Luo and Qi^11^. The slower decay weight set, rather than a sharper decay set, was adopted because smoking cessation treatment is a specialized outpatient service that requires repeated continuation visits; patients may therefore be expected to travel relatively longer distances than for primary care, making a more gradual decay with distance an appropriate assumption.

*Step 1: supply-side evaluation*

The demand population within a defined catchment from healthcare facility j is aggregated with distanceweighted sums to calculate the supply-to-demand ratio Rj. This indicates the capacity of facility j relative to surrounding demand:

R_j_ = S_j_/(ΣP_k_×W1 + ΣP_k_×W2 + ΣP_k_×W3)

where R_j_ is the supply-to-demand ratio of facility j, S_j_ is the supply capacity of facility j, P_k_ is the demand population (smoking rate-adjusted) at point k reachable from facility j, and W1–W3 are the distance decay weights.

*Step 2: demand-side evaluation*

The supply-to-demand ratios R_j_ of reachable healthcare facilities within a defined catchment from each point i were summed with distance weighting to calculate the accessibility score (AS). This indicates the total healthcare resources available to the residents at point i:

AS_i_ = (ΣR_j_×W1 + ΣR_j_×W2 + ΣR_j_×W3) × 10^6^

where AS_i_ is the accessibility score at point i (per million smokers) and R_j_ is the supply-to-demand ratio of facility j reachable from point i.

Travel times were calculated using Network Analyst in ArcGIS PRO software. Meshes with travel times of <5 min were treated as missing, owing to limitations in road data precision.

### Disparity quantification and cluster analysis

Gini coefficients were calculated to quantify intraregional disparities within secondary medical areas. Although Gini coefficients are widely used in economics to measure income distribution inequality, they have also been applied as disparity indicators in healthcare access research using the E2SFCA method^12^. Gini coefficients range from 0 to 1, with values closer to 0 indicating smaller disparities and values closer to 1 indicating larger disparities.

Ward’s hierarchical cluster analysis was performed after standardizing the median AS and Gini coefficients for each secondary medical area. The optimal number of clusters was determined using the Calinski-Harabasz index^31^.

Associations between regional blocks and population sizes were examined descriptively^32^ for the identified clusters. The regional blocks were categorized into nine groups: Hokkaido, Tohoku, Kanto, Hokuriku-Koshin, Tokai, Kinki, Chugoku, Shikoku, and Kyushu Okinawa. Population size (in million) was classified into five categories (<0.100; 0.100–0.300; 0.300–0.500; 0.500–1.000; and >1.000). The SCR distribution by cluster was also examined descriptively.

### Ethics

All the data used in this study are publicly available and contain no personally identifiable information. Therefore, ethics committee approval was not required for this study.

### Statistical analysis

For descriptive statistics, continuous variables were presented as medians with ranges, and categorical variables as frequencies and percentages. As this study covered all secondary medical areas nationwide as a complete enumeration, statistical tests were not performed. GIS analysis was conducted using ArcGIS PRO, and statistical analysis was performed using Stata 18.0 SE. Regarding missing data, the 16 secondary medical areas with missing standardized claim ratio (SCR) data for any year during 2018–2022 were excluded from the analysis, leaving 319 areas. For the regional typology, Ward’s hierarchical cluster analysis was applied to the standardized median AS and Gini coefficient of each secondary medical area, and the optimal number of clusters was determined using the Calinski-Harabasz index.

## RESULTS

### Overview

Of the 335 secondary medical areas nationwide, 16 (primarily remote islands and isolated regions) with missing SCR data were excluded, leaving 319 areas for analysis. The number of facilities registered as smoking cessation clinics and hospitals nationwide is 15139. The range of AS was 0–375734.4, and the Gini coefficient was 0.06–0.75.

### Accessibility score distribution

Table 1 shows the distribution of accessibility scores and Gini coefficients by region.

**Table 1:** Distribution of accessibility scores and Gini coefficients by regional block across 319 secondary medical areas in Japan, a nationwide cross-sectional descriptive study using 2025 population and facility data

*Regional block*	*n*	*Median AS* *(range)*	*Median Gini* *(range)*
Hokkaido	17	1020 (287–2095)	0.42 (0.25–0.60)
Tohoku	34	1020 (286–2299)	0.24 (0.17–0.45)
Kanto	65	893 (550–1784)	0.17 (0.06–0.45)
Hokuriku-Koshin	31	1069 (530–5626)	0.23 (0.12–0.55)
Tokai	28	961 (360–1466)	0.22 (0.10–0.55)
Kinki	41	1101 (315–2809)	0.21 (0.07–0.49)
Chugoku	29	1100 (684–1719)	0.27 (0.12–0.46)
Shikoku	15	1525 (898–2190)	0.33 (0.16–0.53)
Kyushu-Okinawa	59	1105 (296–1735)	0.24 (0.12–0.75)
Total	319	1040 (286–5626)	0.22 (0.06–0.75)

AS: accessibility score (per million smokers), calculated using the enhanced two-step floating catchment area (E2SFCA) method. The Gini coefficient quantifies intraregional disparity in accessibility, ranging from 0 (no disparity) to 1 (maximum disparity). Of the 335 secondary medical areas nationwide, 16 with missing claims data for 2018–2022 were excluded, leaving 319 areas.

The median AS was 1040 (range: 286–5626). By regional block, the median AS was relatively high in Shikoku (1525), Kinki (1101), and Kyushu Okinawa (1105), and relatively low in Kanto (893) and Tokai (961). The median Gini coefficient was 0.22 (range: 0.06–0.75). The median Gini coefficient was high in Hokkaido (0.42) and Shikoku (0.33), and low in Kanto (0.17).

**Figure 1:**
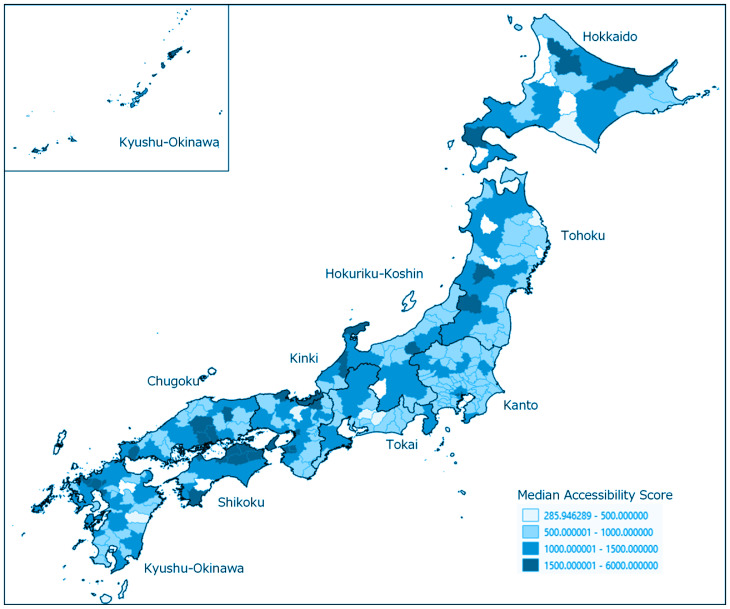
Geographical distribution of median accessibility scores across 319 secondary medical areas in Japan, a nationwide cross-sectional descriptive study using 2025 population and facility data

Figure 1 shows the geographical distribution of the median AS by secondary medical area.

### Cluster analysis

Table 2 presents the cluster characteristics. The Calinski-Harabasz index was the highest among the three clusters, and this three-cluster solution was adopted.

**Table 2:** Characteristics of the three regional clusters identified across 319 secondary medical areas in Japan, a nationwide cross-sectional descriptive study using 2025 population and facility data

*Cluster*	*n (%)*	*Median AS (range)*	*Median Gini (range)*	*Typology*
1	77 (24.1)	859 (286–1774)	0.38 (0.28–0.75)	Low accessibility–High disparity
2	199 (62.4)	1045 (509–1493)	0.20 (0.06–0.31)	Moderate accessibility–Low disparity
3	43 (13.5)	1596 (1355–5626)	0.21 (0.06–0.46)	High accessibility–Low disparity

AS: accessibility score (per million smokers). SMA: secondary medical area. Clusters were derived by Ward’s hierarchical cluster analysis of the standardized median AS and Gini coefficient of each of the 319 SMAs analyzed. The Gini coefficient quantifies intraregional disparity in accessibility, ranging from 0 (no disparity) to 1 (maximum disparity). Clusters 2 and 3 show similar Gini coefficients, differing primarily in their accessibility scores.

Cluster 1 (Low accessibility–High disparity) comprised 77 areas (24.1%), characterized by low AS (median 859) and high Gini coefficients (median 0.38). Cluster 2 (Moderate accessibility–Low disparity) comprised 199 areas (62.4%), characterized by an AS close to the national average (median 1045) and low Gini coefficients (median 0.20). Cluster 3 (High accessibility–Low disparity) comprised 43 areas (13.5%), characterized by relatively high AS (median 1596) and low Gini coefficients (median 0.21). Clusters 2 and 3 had nearly identical Gini coefficients and were primarily distinguished based on their AS levels. The theoretically possible High accessibility–High disparity typology did not emerge naturally.

**Figure 2:**
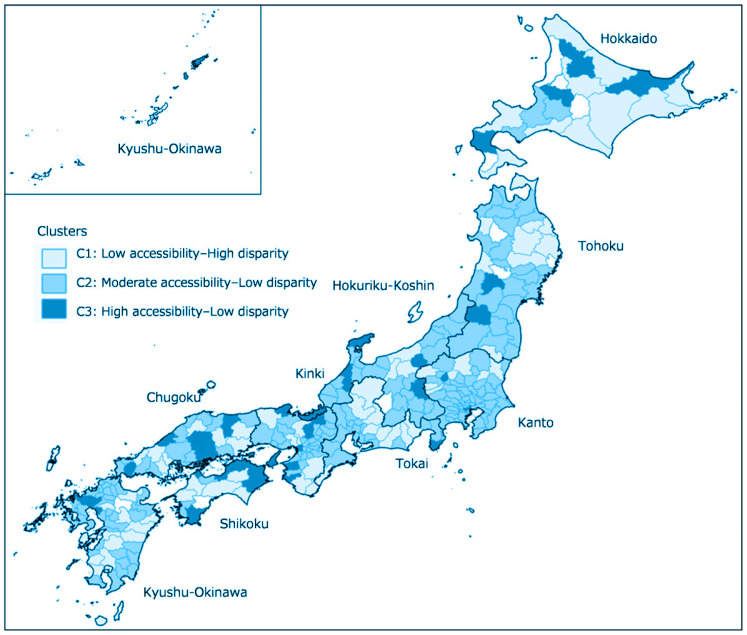
Geographical distribution of the three regional clusters across 319 secondary medical areas in Japan, a nationwide cross-sectional descriptive study using 2025 population and facility data

Figure 2 shows the geographical distribution of the cluster classifications according to the secondary medical areas.

### Geographical and population distribution of clusters

Supplementary file Table 1 shows the cluster distribution based on the regional blocks. In Hokkaido, Cluster 1 (Low accessibility–High disparity) accounted for 58.8%. In Kanto, Cluster 2 (Moderate accessibility–Low disparity) accounted for 84.6%, representing the standard national pattern. In Shikoku, Cluster 3 (High accessibility–Low disparity) was highest at 53.3%.

Table 3 shows the cluster distribution based on the population size.

**Table 3:** Distribution of regional clusters by population size across 319 secondary medical areas in Japan, a nationwide cross-sectional descriptive study using 2025 population and facility data

*Population* *(million)*	*Cluster 1* *Low accessibility–* *High disparity*	*Cluster 2* *Moderate accessibility–* *Low disparity*	*Cluster 3* *High accessibility–* *Low disparity*	*Total*
<0.100	32 (45.7)	21 (30.0)	17 (24.3)	70
0.100–0.300	25 (21.7)	76 (66.1)	14 (12.2)	115
0.300–0.500	10 (17.9)	42 (75.0)	4 (7.1)	56
0.500–1.00	8 (15.7)	38 (74.5)	5 (9.8)	51
>1.00	2 (7.4)	22 (81.5)	3 (11.1)	27
Total	77	199	43	319

Values are presented as n (%); percentages are row percentages within each population-size category.

In small medical areas with populations <0.100 million, Cluster 1 (Low accessibility–High disparity) accounted for 45.7%, and Cluster 3 (High accessibility–Low disparity) accounted for 24.3%. In contrast, in large medical areas with populations >1 million, Cluster 2 (Moderate accessibility–Low disparity) accounted for 81.5%, whereas Cluster 1 accounted for only 7.4%. As population size increased, the proportion of Cluster 2 tended to increase, from 30% at 0.100 million to 81.5% at >1.000 million (Table 3).

### Distribution of treatment utilization by cluster

Table 4 presents the standardized claim ratio (SCR) distribution by cluster (facility–location basis, 2018–2022 average). Areas with missing SCR data for any year were excluded from the analysis (n=16). Values of SCR <90 are below the national average, 90–110 near the national average, and >110 above the national average. In Cluster 3 (High accessibility–Low disparity), the median SCR was 117.4, and areas with SCR >110 accounted for 58.1%. In Cluster 1 (Low accessibility–High disparity), the median SCR was 92.6, and in Cluster 2 (Moderate accessibility–Low disparity), the median SCR was 94.8; areas with SCR <90 accounting for over 40% of both. Cluster 3, with a relatively high AS, tended to have more areas where smoking cessation treatment utilization exceeded the national average.

**Table 4:** Distribution of the standardized claim ratio for nicotine dependence management by regional cluster across 319 secondary medical areas in Japan, a nationwide cross-sectional descriptive study using 2018–2022 claims data

*Cluster*	*n*	*Standardized claim ratio (SCR)*			
		*Median (range)*	*<90*	*90–110*	*>110*
Cluster 1 (Low accessibility–High disparity)	77	92.6 (51.0–242.9)	34 (44.2)	23 (29.9)	20 (26.0)
Cluster 2 (Moderate accessibility–Low disparity)	199	94.8 (46.6–245.1)	85 (42.7)	57 (28.6)	57 (28.6)
Cluster 3 (High accessibility–Low disparity)	43	117.4 (38.1–257.8)	4 (9.3)	14 (32.6)	25 (58.1)

SCR: standardized claim ratio for nicotine dependence management (facility-location basis; mean of fiscal years 2018–2022). SCR is the ratio of observed to expected claims adjusted for population composition: <90, below the national average; 90–110, near the national average; >110, above the national average. Values are presented as n (%); percentages are row percentages within each cluster. Of the 335 secondary medical areas nationwide, 16 with missing SCR data for any year during 2018–2022 were excluded, leaving 319 areas.

## DISCUSSION

This study evaluated accessibility to smoking cessation clinics and hospitals across 319 secondary medical areas nationwide using large-scale geospatial data (422804 mesh points) and the E2SFCA method and identified three regional typologies through cluster analysis. Although geographical approaches in tobacco research have primarily focused on retail environments^6^, this study is novel because it addresses the symmetric question of access to smoking cessation treatment facilities.

The first finding was that clear regional disparities exist in terms of accessibility to smoking cessation clinics and hospitals. The median AS ranged widely from 286 to 5626, and the Gini coefficients ranged widely from 0.06 to 0.75. Accessibility was relatively high in western regions and lower in the metropolitan Kanto and Tokai areas, while intra-regional disparities were comparatively large in Hokkaido and Shikoku and smaller in Kanto.

Second, clear associations were observed between the clusters and regional blocks/population sizes. Cluster 1 (Low accessibility–High disparity) was prevalent in Hokkaido (58.8%) and concentrated in small medical areas (40.2%). In these regions, facilities are limited relative to the vast geographical areas, resulting in large intra-regional disparities in facility access. In contrast, Cluster 2 (Moderate accessibility–Low disparity) was concentrated in the Kanto region (84.6%) and comprised the majority of the medium and large medical areas (70–78%). This represents the standard national pattern, where facilities are relatively evenly distributed, but the population concentration maintains a supply–demand ratio near the national average.

Cluster 3 (High accessibility–Low disparity) was concentrated in Shikoku (53.3%), and the population structure may have contributed to this regional characteristic. Shikoku has a nationally low smoking rate (15.4%, second lowest among the nine regional blocks) and a small total population (3.64 million). Because AS calculated by the E2SFCA reflects facility supply per smoker population, regions with relatively small smoker populations tended to have higher AS. Additionally, Shikoku is geographically compact. Particularly on the Seto Inland Sea side (such as Kagawa Prefecture), the distances to facilities are short, and Gini coefficients tend to be low. However, even within Shikoku, mountainous and remote areas (such as central Kochi) had high Gini coefficients and belonged to Cluster 1, indicating intra-regional heterogeneity. These patterns can be understood descriptively in terms of how the E2SFCA score is constructed. Because the score reflects facility supply relative to the smoker population within the catchment, accessibility tends to be higher where the smoker population is smaller relative to the available facilities. Intra-regional disparity, in turn, depends on how evenly facilities are distributed across the area: where population and facilities are both concentrated, residents tend to share similar accessibility, yielding lower Gini coefficients, whereas in geographically dispersed areas with facilities clustered in a few locations, accessibility varies widely across the area, yielding higher Gini coefficients. The interaction of these two factors – the supply-to-population balance and the spatial evenness of facility distribution – accounts for the co-occurrence of higher accessibility with lower disparity observed across clusters.

Third, examination of SCR distribution by cluster showed that Cluster 3 (High accessibility–Low disparity) had a median SCR of 117.4, with 58.1% of areas having SCR >110. In contrast, Clusters 1 and 2 had median SCR of 92.6–94.8, with over 40% of areas having SCR <90. As this was a cross-sectional ecological analysis, no causal or directional relationship can be inferred; the findings indicate only that, descriptively, the cluster with relatively higher AS also showed higher SCR values.

Whereas geographical tobacco research has concentrated on the tobacco supply side and E2SFCA-based accessibility research has centered on primary care and general hospital services^33^, the present study lies at the intersection of these two strands by quantifying access to cessation treatment itself.

Compared to the limited previous research on geographical access to smoking cessation treatment, a previous study reported differences in cessation treatment utilization rates between rural and urban areas and showed an individual-level association between distance and cessation class participation rates^9,10^. This study complements previous research by describing accessibility indicators and the distribution of smoking cessation treatment utilization at the regional level on a nationwide scale.

The typology suggests that smoking cessation support should be tailored to regional characteristics rather than applied uniformly. Geographical barriers are most pronounced in Cluster 1, where countermeasures must address both the absolute shortage of facilities and the uneven distribution of facilities, whereas Clusters 2 and 3 call for approaches suited to denser, more evenly served settings. These countermeasures should not be mutually exclusive but combined according to local conditions.

The first countermeasure was telemedicine. A systematic review of e-health interventions reported that digital technology-based interventions significantly improved quitting rates^34^. Particularly in small medical areas in the Hokkaido and Tohoku regions belonging to Cluster 1 (Low accessibility–High disparity), new facility development alone is difficult owing to geographical constraints, and telemedicine can be an effective means of overcoming physical distance barriers.

The second countermeasure is the use of community pharmacy-based cessation programs. A Cochrane review showed that pharmacy-based smoking cessation support interventions significantly improved smoking cessation rates^35^. A systematic review also reported the effectiveness of pharmacy-based smoking cessation support (OR=2.56)^36^. Pharmacies are often located in more accessible places than medical facilities; particularly in large medical areas in the Kanto region belonging to Cluster 2 (Moderate accessibility–Low disparity), they can be an effective means of increasing access points for smoking cessation support in response to population concentration. These countermeasures are not mutually exclusive and may be effective for any typology. Flexible combinations tailored to local circumstances are important.

### Strengths and limitations

This study has the following strengths. First, this is the first smoking cessation facility accessibility evaluation using large-scale geospatial data from approximately 400000 mesh points nationwide, comprehensively analyzing entire regions and complementing existing individual-level studies. Second, the E2SFCA method, which considers both supply and demand, provides a more realistic evaluation of accessibility than simple facility count or distance. Third, by evaluating accessibility levels and intra-regional disparities (Gini coefficients) and conducting a cluster analysis based on both indicators, this study provides useful insights for policy development. Fourth, by descriptively examining the association with SCR based on nationwide claims data, this study obtained findings suggesting a relationship between geographical accessibility and smoking cessation treatment utilization.

This study has several limitations. First, the facility data are based on registration information and cannot distinguish between facilities that actively provide smoking cessation (clinics and hospitals). Second, this was a cross-sectional descriptive study and did not verify causal relationships between accessibility and utilization. Third, the SCR is aggregated based on facility location and does not directly reflect utilization based on patient residence. However, smoking cessation clinics and hospitals require continuous visits, and patients are unlikely to visit facilities far from their residences. Additionally, claim counts include continuation visits, and regional differences in completion rates may affect the SCR. Fourth, the smoking rates used for demand estimation are sex- and age-specific values based on national surveys and do not account for regional differences in smoking rates. Fifth, the supply capacity of each facility was approximated using the average number of physicians by facility type, rather than facility-level indicators of actual smoking cessation service capacity. This approach does not capture differences in the number of cessation sessions provided, the presence of trained staff, opening hours, or whether cessation treatment is actively delivered, and it may therefore overestimate the effective availability of cessation services. Sixth, the 16 secondary medical areas excluded because of missing SCR data were primarily remote islands and isolated regions, which may have poorer geographical access; their exclusion could lead to an underestimation of the true extent of geographical disparities. Seventh, the analysis assumed travel by automobile and walking and did not incorporate public transportation, which plays an important role in urban Japan; transportation access may also vary with age, rurality, and socioeconomic conditions. Eighth, the catchment-area thresholds and distance-decay weights were based on parameters from previous E2SFCA research, and a systematic sensitivity analysis of alternative parameter choices was not conducted; however, as this study is descriptive in nature and focuses on relative geographical patterns of accessibility across regions rather than on absolute accessibility values, the influence of specific parameter values on the overall conclusions is likely to be limited. Finally, the accessibility measure reflects potential spatial accessibility and cannot capture service quality, appointment availability, or actual patient mobility patterns. Taken together with the descriptive and ecological design of this study, these limitations constrain the generalizability of the findings: the results characterize relative geographical patterns of potential accessibility at the regional level and should not be generalized to individual-level access, to causal relationships between accessibility and treatment utilization, or to settings with markedly different healthcare or transportation systems.

### Future research

Future research directions include longitudinal studies using individual-level data to verify associations between accessibility and smoking cessation treatment initiation, completion, and cessation success, evaluation of the impact of expanded telemedicine and pharmacy-based smoking cessation support programs on accessibility, and application of the methodology used in this study to other countries for international comparison of the FCTC Article 14 implementation status.

## CONCLUSIONS

This study demonstrated substantial geographical disparities in accessibility to smoking cessation treatment facilities across secondary medical areas in Japan and identified three distinct regional typologies. Regions with higher accessibility tended to exhibit lower intra-regional disparities, and the cluster with the highest accessibility also showed higher treatment utilization based on nationwide claims data. While geographical research in tobacco control has concentrated on retail environments, this study addresses the underexplored question of access to cessation services. For low-accessibility areas where establishing new clinics is geographically difficult, approaches that do not depend on the physical placement of clinics – such as telemedicine and community pharmacy-based cessation support– may help reduce geographical barriers. These descriptive findings suggest that, for the implementation of WHO FCTC Article 14, the equitable geographical distribution of services within regions, alongside the overall number of facilities, may be a relevant consideration; further studies of different designs are needed to establish this more firmly. The analytic framework presented here is transferable to other countries seeking to evaluate their cessation service infrastructure.

## Supplementary Material



## Data Availability

Data sharing is not applicable to this article as no new data were created.
