# Supplementary material for: Geographical accessibility to smoking cessation treatment facilities across 335 medical areas in Japan: A nationwide cross-sectional descriptive study using large-scale geospatial data
Source: Tob Induc Dis. 2026 Jul 18;24:10.18332/tid/222677. doi: 10.18332/tid/222677 (PMC13386697; doi:10.18332/tid/222677)
Supplement: Supplementary file 1 [file TID-24-115-s1.pdf]

Tobacco Induced Diseases

Supplementary file

© 2026 Egashira Y. and Watanabe R.

DOI:
10.18332/tid/222677

The content has been provided by the author(s) and has not been reviewed, verified, or endorsed by European Publishing. It may not have undergone peer review. The views, opinions, and recommendations expressed are solely those of the author(s) and do not necessarily reflect the position of European Publishing. European Publishing accepts no responsibility or liability for any consequences arising from the use of, or reliance on, this content.

Supplementary file

Supplementary Table S1. Cluster Distribution by Regional Block

**Regional Block**	**Cluster 1** Low access–High disparity	**Cluster 2** Moderate access–Low disparity	**Cluster 3** High access–Low disparity	**Total**
Hokkaido	10 (58.8)	3 (17.6)	4 (23.5)	17
Tohoku	9 (26.5)	23 (67.6)	2 (5.9)	34
Kanto	7 (10.8)	55 (84.6)	3 (4.6)	65
Hokuriku-Koshin	3 (9.7)	23 (74.2)	5 (16.1)	31
Tokai	9 (32.1)	18 (64.3)	1 (3.6)	28
Kinki	7 (17.1)	26 (63.4)	8 (19.5)	41
Chugoku	9 (31.0)	13 (44.8)	7 (24.1)	29
Shikoku	5 (33.3)	2 (13.3)	8 (53.3)	15
Kyushu-Okinawa	18 (30.5)	36 (61.0)	5 (8.5)	59
Total	77	199	43	319

Values are presented as n (%). Cluster 1: low access-high disparity; Cluster 2: moderate access-low disparity; Cluster 3: high access-low disparity.
